# Age-Dependent Expression of MyHC Isoforms and Lipid Metabolism-Related Genes in the Longissimus Dorsi Muscle of Wild and Domestic Pigs

**DOI:** 10.3390/ani9010010

**Published:** 2018-12-28

**Authors:** Milka Vrecl, Marko Cotman, Matjaž Uršič, Marjeta Čandek-Potokar, Gregor Fazarinc

**Affiliations:** 1 Veterinary Faculty, Institute of Preclinical Sciences, University of Ljubljana, Gerbičeva 60, 1000 Ljubljana, Slovenia; milka.vrecl@vf.uni-lj.si (M.V.); marko.cotman@vf.uni-lj.si (M.C.); matjaz.ursic@vf.uni-lj.si (M.U.); 2 Agricultural Institute of Slovenia, Hacquetova ulica 17, 1000 Ljubljana, Slovenia; meta.candek-potokar@kis.si

**Keywords:** skeletal myofibers, postnatal transformation, quantitative PCR, histochemistry, MyHC isoforms, lipid metabolism-related genes

## Abstract

**Simple Summary:**

This comparative study found that postnatal myofiber differentiation in the longissimus dorsi muscle of wild pigs was accelerated and directed toward oxidative myofibers in which the *MyHC-I, -IIa* and -*IIx* genes were well expressed. In domestic pigs, the transformation was directed toward glycolytic myofibers in which the *MyHC-IIb* gene transcript was abundant. The myofiber succinate dehydrogenase (SDH) activity and lipid content reflected expression patterns of the MyHCs and not genes involved in lipid uptake and utilization (*PGC-1α, PPARγ, LPL*, and *CPT-1B*). MyHC myofiber characteristics reflect the effects of selection pressure and rearing conditions on the growth and lean meat content and may be valuable for establishing a balanced breeding scheme focused on improving meat quality traits.

**Abstract:**

This study aimed to compare age-dependent changes in the relative expression of genes encoding myosin heavy chain (MyHC) isoforms and selected lipid metabolism-related genes in the longissimus dorsi muscle of wild pigs (WPs) and domestic pigs (DPs). Muscles sampled from postnatal day one as well as three-week-old and two-year-old animals were used in quantitative polymerase chain reaction (qPCR) assays, histological evaluations of succinate dehydrogenase (SDH) activity, and intra-myofiber lipid (IMFL) assessment. Expression of the MyHC isoforms displayed the most extensive age- and breed-dependent changes within the first three postnatal weeks. The *MyHC_embry_* level decreased significantly faster in the WPs than in the DPs. The relative *MyHC-I* and *-IIa* expression was significantly higher in the WPs, and *MyHC-IIb* was substantially higher in the DPs. The differences in *MyHC* expression corroborated the number of SDH-positive myofibers and IMFLs. Expression of the peroxisome proliferator-activated receptor gamma coactivator 1 alpha (PGC-1α), peroxisome proliferator-activated receptor gamma (PPARγ) and lipoprotein lipase (LPL) genes displayed only age-related variations. In summary, the evidence is provided for accelerated postnatal myofiber transformation directed towards oxidative myofibers in WPs. The SDH activity/staining intensity largely reflected the expression of MyHCs, and not genes involved in lipid uptake and utilization.

## 1. Introduction

In modern pig breeds, genetic selection is focused on achieving fast growth and leanness of the carcass. This focus has resulted in myofiber hypertrophy, an increased proportion of IIb myofibers, and a general switch toward more glycolytic metabolism in the musculature of modern pig breeds [1]. However, these same characteristics also have a negative impact on meat quality [2,3]. This incompatibility between traits associated with meat quality and growth performance has prompted studies of different pig breeds and rearing conditions to better understand the balance among growth performance, muscularity, and meat quality [1,4]. Myosin heavy chain isoforms (MyHCs) are the principal myofibrillar proteins that control myofiber contractile properties. As such, they are the main determinants of muscle phenotypes. The locomotor skeletal muscles of adult pigs express one slow (*MyHC-I*) and three fast (*MyHC-IIa, -IIx* and *-IIb*) MyHC isoforms [5]. These isoforms are encoded by a multigene family that is organized into two clusters: Cardiac (*MyHC slow/b* and *α*) and skeletal (MyHC embryonic, 2a, 2x, 2b and perinatal) [6]. The MyHC expression patterns largely define the myofiber phenotype as well as the diversity of the metabolic properties, myofiber size, and glycogen and lipid contents (reviewed by Lefaucheur [2]). Type I and IIa myofibers are predominantly oxidative, use lipids as their main energy source, and perform sustained contractions, whereas type IIb myofibers use glycogen as their primary energy source and perform quick and strong contractions. Type IIx myofibers represent metabolically intermediate types that lie between type IIa and IIb myofibers [2]. These characteristics are acquired as a result of time-dependent developmental changes in MyHCs expression patterns and the myofiber metabolic profile. The typical arrangement of myofiber types in the fasciculus muscle of adult pigs is determined primarily by prenatal development of the primary and secondary myofibers. During the prenatal period, primary myofibers express *MyHC-I*, whereas fast secondary myofibers primarily express developmental (embryonic/perinatal) MyHCs. Then, during the early postnatal period, the expression of slow *MyHC-I* increases and that of developmental MyHCs diminishes in the secondary myofibers that surround the centrally positioned primary myofibers [7]. Expression of the developmental forms of MyHC decreases toward the end of gestation when these forms are replaced by the adult fast MyHCs in the following sequence: Embryonic/perinatal > *IIa* > *IIx* > *IIb. MyHC-IIa* and *-IIx* are already co-expressed in secondary myofibers at birth, whereas *MyHC-IIb* first appears during the early postnatal period [8]. Regarding metabolic phenotypes, all myofibers are oxidative at birth. However, in parallel with the transition to the expression of adult MyHCs that occurs during the first postnatal weeks, a metabolic switch also occurs as they differentiate into oxidative, oxidative-glycolytic, or glycolytic myofibers [9]. In the pig, the oxidative myofiber types that express *MyHC-I, -IIa,* and *-IIx* are also associated with desirable meat quality properties, such as the water-holding capacity, pH, and tenderness (reviewed by Listrat et al. [10]). The ratio of preferentially oxidative myofiber types is higher in wild pigs (WPs) and some indigenous pig breeds than in the highly selected modern domestic pig (DP) breeds [11,12,13]. A recent comparative study of young pigs of the Iberian and conventional breeds showed that under identical nutrition and management conditions, the Iberian pigs had a higher intramuscular fat content and oxidative metabolism in the longissiumus dorsi muscle [14].

A very important indicator of meat quality that is highly correlated with the meat tenderness, water-holding capacity, flavor, and juiciness is the intramuscular fat content (reviewed by Lefaucheur (2010) [2]). Hence, recent studies have focused on genes that are important for the different lipid metabolic processes in myofibers. One of these genes is peroxisome proliferator-activated receptor gamma coactivator 1 alfa (*PGC-1α*), which regulates a set of downstream target genes and plays a crucial role in myofiber metabolism and type maturation and can even induce myofibers to transform from the fast to the slow type [15]. In vitro evidence suggests that this gene also plays a role in myoblast differentiation [16]. Additionally, other lipid metabolism-related genes, especially those involved in lipogenesis (e.g., peroxisome proliferator-activated receptor gamma, *PPARγ*), fatty acid uptake (e.g., lipoprotein lipase, *LPL*), and fatty acid oxidation (carnitine palmitoyltransferase-1B, *CTP-1B*), have attracted research interest in pig muscle tissues because of their potential association with meat quality [12,13,17].

To understand better the mechanisms behind meat quality deterioration related to domestication and selection pressure, age-related changes in *MyHC* expression profiles and lipid metabolism were studied in wild and modern highly selected pigs using two genetically distant European pig breeds reared in contrasted conditions. In our previous study, we performed enzyme-immunohistochemical analysis in WPs and DPs, and our results suggested distinct differences in the maturation of different myofiber types and their hypertrophic potential [11]. However, qPCR analysis can more sensitively/accurately identify MyHC isoforms at the mRNA level and provide additional important information about muscle development. Moreover, because many qualitative traits of meat are associated with the intramuscular fat content, enhancing understanding of the genetic basis of lipid metabolism is a topic of interest. Therefore, the main objective of the present comparative study was to evaluate age-dependent changes in the relative expression of mRNAs encoding MyHCs, reflecting the contractile phenotype and selected genes that encode proteins involved in lipid metabolism in WPs and DPs, and to provide additional information about their potential predictive value for optimizing meat quality traits. 

## 2. Materials and Methods 

### 2.1. Animals and Muscle Samples

Muscle samples were collected from the longissimus dorsi muscle of European WPs (*Sus scrofa scrofa*) and DPs (Large White) at three different ages as previously described [11]. Briefly, an approximately one cubic centimeter muscle sample was taken from the central part of the longissimus dorsi muscle at the level of the last rib within 24 h post-mortem, frozen in liquid nitrogen and preserved at −80 °C before further processing. The muscle samples were obtained from 1-day-old piglets (24–48 h post-partum, 4 WPs (body mass from 0.96 to 1.28 kg) and 4 DPs (body mass from 1.12 to 1.46 kg)), 3-week-old pigs (21–23 days old, 5 WPs (body mass from 1.92 to 2.43 kg) and 5 DPs (body mass from 3.72 to 4.11 kg)) and approximately 2-year-old adult pigs (6 WPs (body mass between 46 and 65 kg) and 6 DPs (body mass between 185 and 200 kg)). Only female pigs were included in the study. The DPs were raised and slaughtered according to standards for farm and slaughter procedures, respectively, and the WPs were reared in a large hunting enclosure (>1,000 ha) with a confined population of 100 to 140 animals (i.e., ten boars, 20 to 25 sows, approximately 35 yearlings, and approximately 70 piglets). WP piglets that were found dead (drained nest), injured due to overlaying or abandoned by the sow were collected by the professional hunter that monitored pregnant sows and the status of the farrowing nests on a daily basis. WP piglets were collected during the spring over a period of two years and selected for the analysis based on age, body mass, and gender. Adult WPs were shot based on regular annual bagging during the hunting season, and the professional hunter estimated their ages. All animal procedures were performed in accordance with the protocols described in the Slovenian Law of Animal protection and were not subject to ethical protocols according to Directive 2010/63/EU (2010). The veterinary faculty is an approved establishment by the Veterinary Administration of the Republic of Slovenia (Approval No. SI B 07-22-25) for use of animal by-products C2 (Category 2 1069/2009/ES) and C3 (Category 3 1069/2009/ES) for research purposes. 

### 2.2. RNA Isolation, cDNA Synthesis and Quantitative Polymerase Chain Reaction (qPCR)

Total RNA was extracted from the collected muscle samples using the RNeasy Fibrous Tissue Mini Kit (Qiagen, Stockach, Germany) according to the manufacturer’s protocol, including an on-column DNase digestion step. RNA sample quality control was performed by Novogene Co., Ltd. (Wan Chai, Hong Kong) using the following methods: (i) NanoDrop^TM^ (Thermo Fisher Scientific, Waltham, MA, USA), agarose gel electrophoresis and Agilent 2100 for the preliminary quantitation, testing of RNA degradation/potential contamination and RNA integrity check and quantitation, respectively. RNA samples that passed the quality control analysis were used in the subsequent steps. Five out of the 30 RNA samples, including four extracted from the WP muscle samples, were excluded due to RNA degradation (i.e., a RNA integrity number (RIN) lower than 6, which was stringent considering that a RIN higher than 5 was suggested to represent good total RNA quality for downstream application [18]). One μg of each RNA sample was reverse-transcribed into cDNA using an RT² First Strand Kit (Qiagen, Stockach, Germany) according to the manufacturer’s protocol. Primers and fluorescent 6-FAM dye-labeled minor-groove-binder probes/pre-developed assays were acquired from Applied Biosystems (Thermo Scientific GmbH, Vienna, Austria) and used to detect the MyHC isoforms and selected lipid metabolism-related genes (shown in Table 1). The qPCR was performed using the TaqMan universal PCR Master Mix (Applied Biosystems, Thermo Scientific, GmbH, Vienna, Austria) in the AbiPrism 7000 Sequence Detection System. The cDNA preparations were diluted 10-fold prior to the qPCR analysis. PCR amplification was carried out under the following conditions: One cycle of 50 °C for 2 min, one cycle of 95 °C for 10 min and 45 cycles of 15 sec at 95 °C and 1 min at 60 °C. Each reaction (in a final reaction volume of 20 μL) was run in triplicate. The results were calculated from a threshold cycle (Ct), which was the cycle number at which the PCR product crossed the detection threshold. A Ct greater than 40 was defined as the cut-off (the point at which a gene’s expression was undetected), as previously reported [19]. The eukaryotic 18S ribosomal (r) RNA (18S rRNA) was tested as an endogenous control because it was found previously to be stably expressed in samples containing equal amounts of cDNA [20]. The effect of age/breed on 18S rRNA expression was validated using the 2^−ΔCt^ equation adopted from Livak and Schmittgen, 2001 [21] as follows: ΔCt = (Ct_agex_-Ct_day1_). No significant relationship was found between age/breed and 18S rRNA expression (Supplementary Figure S1); therefore, 18S rRNA was used as the internal control to normalize the data using the comparative Ct method (ΔCt); ΔCt = Ct(target gene)—Ct(18S rRNA) and derived ΔCt are shown in Supplementary Table S1. The 2^−ΔΔCt^ method [21] was used to present the relative fold changes in the expression of the examined genes between the WPs and DPs, and to compare age/breed-dependent patterns in gene expression relative to expression in the 1-day old wild piglets. The PCR efficiency of the studied genes was >90% and was determined from standard curves composed of four 10-fold dilutions of the cDNAs. Control experiments were performed without reverse transcription (RT) to demonstrate that no genomic DNA contamination was present. 

### 2.3. Histochemistry 

The same muscle samples used in the qPCR analysis were also used to obtain transverse cryosections (10 μm), which were stained for succinate dehydrogenase (SDH) activity and the amount of lipids in the myofibers. The cryosections were cut using a cryostat Leica CM 1800 at −17 °C and mounted onto (3-aminopropyl) triethoxysilane (APES) coated slides. The oxidative capacity of the myofibers was determined by analyzing the mitochondrial SDH activity as previously described [22]. The sections were dehydrated and mounted using Synthetic Mountant (Shandon, CA, USA). 

An optimized oil red O staining protocol developed by Koopman et al. [23] was used to demonstrate the intra-myofiber lipids (IMFLs). Briefly, air-dried and thawed transverse cryosections of muscle tissue were fixed in 3.7% formaldehyde for 60 min, rinsed with deionized water and then stained with oil red O diluted in triethyl phosphate for 30 min. The sections were rinsed with tap water and mounted using the anti-fade ProLong^®^ Gold reagent (Molecular Probes, Leiden, The Netherlands). 

### 2.4. Histological Examination 

A Nikon Microphot FXA microscope equipped with a DS-Fi1 camera and the NIS Elements D.32 imaging software (Nikon instruments Europe B.V., Badhoevedorp, The Netherlands) and a Leica multispectral confocal laser microscope (Leica TCS NT, Heidelberg, Germany) were used to examine the cryosections. Oil red O-stained lipids were detected under the confocal microscope using an excitation laser line at 543 nm (helium-neon). Optical sections (1.0 µm) were acquired, and images were produced using an 8-fold frame averaging a 1024 × 1024 pixel resolution. Representative images were presented using Adobe Creative Cloud (Adobe Inc., San Jose, CA, USA). 

### 2.5. Statistical Analysis

Data were subjected to two-way analysis of variance. The latter included fixed effects of age (day 1, 3 weeks, and 2 years), breed (WP vs. DP) and their interaction. The analyses were performed using the Sigma Plot 11.0 statistical software (Systat Software Inc., San Jose, CA, USA). Means were compared with Tukey’s multiple comparisons tests for unequal sample sizes and differences considered statistically significant when *p*-value was <0.05.

## 3. Results

### 3.1. MyHC Isoform Expression Patterns

**Figure 1:**
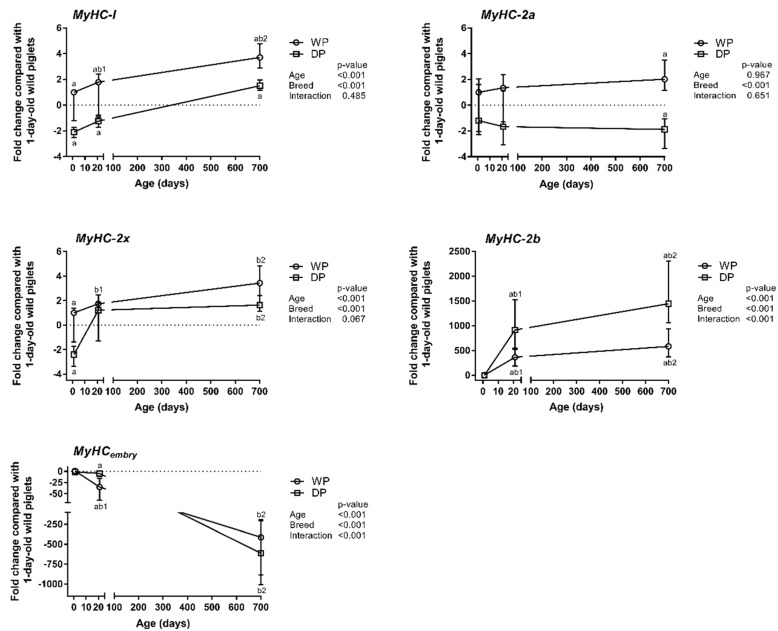
Age- and breed-dependent changes in MyHC isoform expression in the longissimus dorsi muscle of wild pigs (WPs) and domestic pigs (DPs). Shown are the mean fold changes with upper and lower limits in MyHCs expression for WPs and DPs compared with the expression in 1-day-old wild piglets. A fold-change value less than 1 was replaced by a negative inverse of the original fold-change value. Upper and lower limits were calculated for each fold-change value using the standard error of the mean. Effect (*p*-values) for age, breed, and their combined effect (age × breed interaction) were derived by two-way analysis of variance. Differences were considered significant at *p* < 0.05 (Tukey’s multiple comparisons tests). ^a^-significantly different between breeds within an age group; ^b1^-significantly different between 1-day-old wild piglets and 3-week-old pigs; ^b2^-significantly different between 1-day-old wild piglets and 2-year-old pigs.

The relative mRNA expression levels of the MyHC isoforms were assessed in the longissimus dorsi muscle of 1-day-old piglets and 3-week-old and 2-year-old WPs and DPs using qPCR. The results are presented as the mean fold change of differences in MyHCs expression relative to that of the 1-day-old wild piglets (Figure 1). In the 1-day-old piglets (day 1), we found that although the MyHC_embry_, -I, -IIa and -IIx isoform mRNAs were abundant in both breeds, the MyHC-I and -IIx levels were significantly lower in the DPs than in the WPs. The relative MyHC-IIb expression level was very low at this age in both the WP and DP piglets (Figure 1 and Supplementary Table S1). In both breeds, MyHC-IIb and MyHC_embry_ exhibited the most striking age-dependent differences. However, the increase in the MyHC-IIb level was substantially more dramatic in the DPs than in the WPs, whereas the decrease in the MyHC_embry_ level occurred significantly faster in the WPs and then reached the detection limit in the adult animals of both breeds. MyHC-IIb and MyHC_embry_ were the only MyHC isoforms for which interactions between age and breed was observed. A moderate but significant increase in the relative expression of MyHC-I and -IIx was observed only in the WPs, and the difference in MyHC-I expression was also age-dependent. MyHC-IIa expression tended to increase in the WPs and decrease in the DPs, but the breed-dependent difference was observed only in adult animals (Figure 1, Supplementary Table S1).

### 3.2. Expression Patterns of Lipid Metabolism-Related Genes

**Figure 2:**
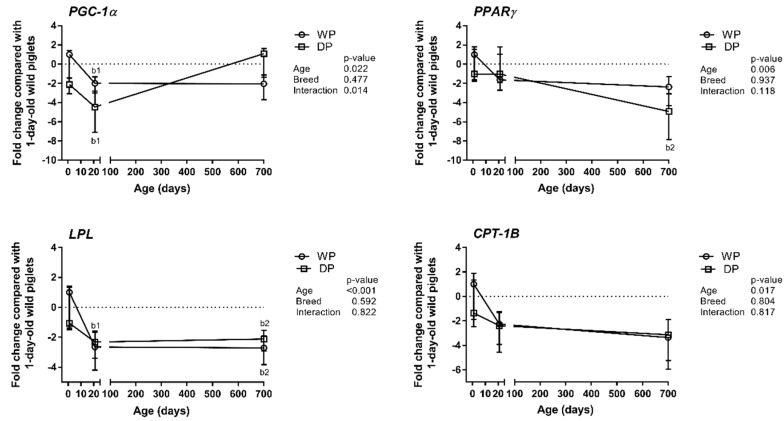
Age- and breed-dependent changes in lipid metabolism-related gene (*PGC-1α*, *PPARγ*, *LPL*, and *CPT-1B*) expression in the longissimus dorsi muscle of wild pigs (WPs) and domestic pigs (DPs). Shown are the mean fold changes with upper and lower limits in lipid metabolism-related genes expression for WPs and DPs compared with the expression in 1-day-old wild piglets. A fold-change value less than 1 was replaced by a negative inverse of the original fold-change value. Upper and lower limits were calculated for each fold-change value using the standard error of the mean. Effect (*p*-Values) for age, breed, and their combined effect (age × breed interaction) were derived by two-way analysis of variance. Differences were considered significant at *p* < 0.05 (Tukey’s multiple comparisons tests). ^a^-significantly different between breeds within an age group; ^b1^-significantly different between 1-day-old wild piglets and 3-week-old pigs; ^b2^-significantly different between 1-day-old wild piglets and 2-year-old pigs.

The results obtained for the selected lipid metabolism-related genes (i.e., PGC-1α, PPARγ, LPL, and CPT-1B) are summarized in Figure 2. The expression levels of these genes were not significantly influenced by breed. PGC-1α showed a decreasing trend only in the 1-day-old domestic piglets that did not reach statistical significance (*p* = 0.056), (Figure 2). However, PGC-1α, PPARγ, and LPL displayed some age-related variations. Specifically, the relative expression level of PGC-1α decreased during the first three postnatal weeks and was 2-fold and 4-fold lower in the 3-week-old WPs and DPs, respectively, than in the 1-day-old wild piglets. Then, it increased again in the DPs and reached a level comparable to that in the 1-day-old wild piglets. The relative expression levels of PPARγ and LPL decreased with age in the WPs and DPs. The significant decrease in PPARγ expression observed in the DPs occurred only after the piglets reached three weeks of age, whereas the decline in the expression of LPL occurred during the first three postnatal weeks. The relative expression level of CPT-1B displayed neither breed- nor age-specific changes (Figure 2). 

### 3.3. Histological Evaluation

**Figure 3:**
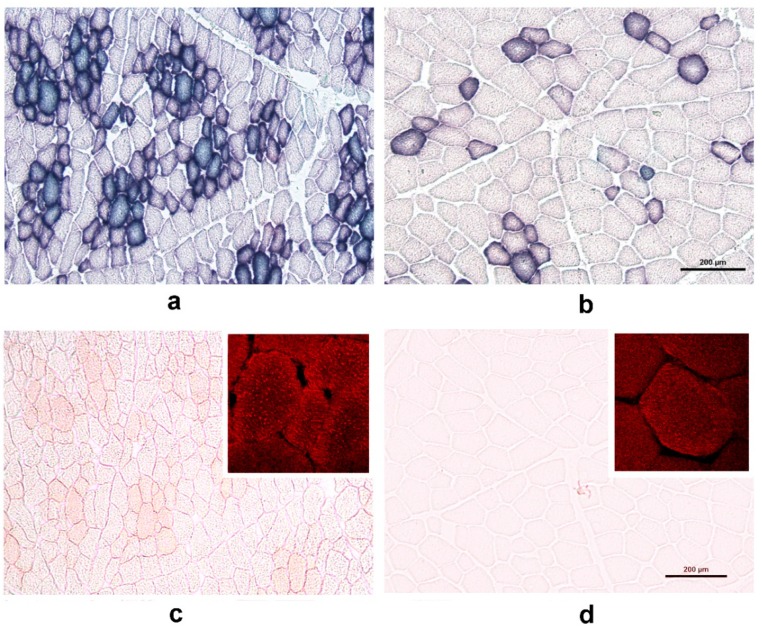
Transverse cryosections of the longissimus dorsi muscle of a 2-year-old wild pig (WP; panels **a** and **c**) and a 2-year-old domestic pig (DP; panels **b** and **d**) were stained for succinate dehydrogenase (SDH) activity (panels **a** and **b**) and the accumulation of neutral lipids within myofibers (using oil red O, panels **c** and **d**). Note that SDH-positive myofibers were more numerous in the WPs than in the DPs (*cf.* panels **a** and **b**) and that the SDH activity pattern largely overlapped with intense oil red O staining in the WPs (*cf.* panels **a** and **c**). In the DPs, oil red O staining was too faint to analyze the intra-myofiber lipids (IMFLs) (panel **d**). The difference in the staining intensity of oil red O-positive fibers between the WPs and DPs was also examined using confocal microscopy (panels **c** and **d**; inserts). Note that fluorescence signals indicative of IMFL droplets (red) were more intense in the WPs than in the DPs. Scale bar = 200 µm (also valid for the WPs).

We performed enzyme histochemistry assays for SDH activity and oil red O staining (Figure 3). The results revealed that SDH-positive myofibers were more numerous in the adult WPs than in the adult DPs. In the WPs, the myofibers exhibiting the most SDH activity largely overlapped with those that were most intensely stained with oil red O, which indicated IMFL accumulation (cf. panels a and c). In the DPs, the oil red O staining was very faint; however, we were able to show that IMFLs were present in individual myofibers in the DPs using confocal microscopy (inserts in Figure 3c,d). 

## 4. Discussion

In this comparative study, we evaluated age-dependent changes in relative mRNA expression of *MyHCs* and selected lipid metabolism-related genes in the longissimus dorsi of WPs and DPs. The relative abundance of *MyHC* transcripts and the relationships among them have previously been studied in diverse pig breeds [3,8,12,13,20,24,25,26,27]. However, only a subset of these studies has explored temporal/developmental changes in *MyHC* expression [8,12,25,28]. Most of these studies compared commercial and indigenous pig breeds [12,13,20,26], and to date, studies involving WPs used only enzyme-immunohistochemical approaches to classify myofiber types [1,4,11,29].

### 4.1. Contractile Phenotype—MyHCs Expression Patterns

Analyses of *MyHC* mRNA expression profiles have been largely in agreement with enzyme-immunohistochemistry analyses of myofiber type compositions [11]. A positive correlation between the relative abundance of each individual *MyHC* mRNA and its corresponding protein in the longissimus dorsi muscle has also been demonstrated in growing commercial crossbred pigs [27]. These results support the hypothesis that *MyHC* genes are transcriptionally regulated in porcine skeletal muscles [3,9]. We observed that the most obvious changes in the *MyHC* transcript levels in both breeds occurred during the first three postnatal weeks. From day one to three weeks postnatal, the order of *MyHC* transcript levels changed as follows: *IIx* > *IIa* > *I* > *embry* > *IIb* to *IIx* > *IIb* > *IIa* > I > *embry*. Although this pattern of postnatal transitions was the same in both breeds, the quantitative changes were substantially different between them. *MyHC_embry_* expression disappeared approximately 10-fold faster in the WPs than in the DPs, whereas *MyHC-IIb* increased to substantially higher levels in the DPs. *MyHC_embry_* is one of the developmental MyHCs that predominate during gestation. Developmental MyHCs are subsequently replaced by adult fast MyHCs (i.e., MyHC-IIa, -IIx and -IIb) in the late gestation/early postnatal period [19]. Thus, the *MyHC_embry_* expression level reflects muscle maturity. *MyHC_embry_* was also reported in 6-week-old DPs [19] and could be associated with slower muscle maturation in these pigs. The higher *MyHC-IIx* expression level observed in the 1-day-old wild piglets additionally confirmed that myofiber maturation and the substitution of developmental with adult MyHC was accelerated during the first post-partum hours in 1-day-old wild piglets.

In addition to age, genes can be up- or down-regulated by a variety of triggering factors, such as nutrition, hormones, and physical activity. Different intrinsic and/or extrinsic insults during gestation and the early postnatal period may have an especially prolonged and substantial influence on MyHC expression and muscle characteristics [30]. The *MyHC-I* expression level was higher in 1-day-old wild piglets than in 1-day-old domestic piglets, perhaps as a consequence of an accelerated transformation from fast to slow MyHCs in the so-called hybrid I/II myofibers during the first hours post-partum. This effect could have been caused by (i) exposure to cold after birth and consequential shivering thermogenesis [31], (ii) a higher *PGC-1α* expression level in the WPs than in the DPs, which is a well-documented cold- and exercise-induced coactivator [32] and supports its role in inducing the transition of myofiber types from fast to slow [15], (iii) limited intrauterine nutrition [33] and iv) undernutrition during the first postnatal days [34]. The *MyHC-I* mRNA abundance subsequently increased in both breeds, but its expression was substantially higher in the WPs than in the DPs in all age groups. The results regarding the *MyHC-I* mRNA expression pattern observed in the present study appear to contradict those in another report, in which the *MyHC-I* mRNA levels were found to decrease with age in commercial pigs [27]. However, in the aforementioned study [27], different age groups were examined; the authors used 7-day old piglets instead of 1-day old piglets. Hence, the relative abundance of *MyHC-I* may increase during the first postnatal week. Furthermore, long-lasting physical activity can also induce myofiber transformation as follows: *IIb* > *IIx* > *IIa* > *I* [2]. These factors, together with exposure to environmental temperatures, which are lower during the greater part of the year for WPs (reared outdoors) than for DPs, were probably the main external factors that could explain the expression trend of MyHCs towards slow-twitch (I) and fast-twitch oxidative (*IIa*) myofibers. The substantially higher *MyHC-IIb* level in the DPs corroborates its abundance in the skeletal muscles of modern pig breeds selected for leanness and high growth performance [5]. At the same time, the *MyHC-IIb*-positive myofibers of DPs have a larger cross-sectional area (CSA) than those of WPs [11]; additionally, their CSA generally is larger than that of type I fibers, and both their abundance and diameter are negatively correlated with meat tenderness [2]. Therefore, the rise in the slow-twitch oxidative myofiber content could be regarded as a promising option to improve the sensory quality of pork [2,35].

### 4.2. Expression Patterns of Lipid Metabolism-Related Genes—Metabolic vs. Contractile Phenotype

Although the *MyHC* mRNA expression patterns have been suggested to be good indicators of meat quality characteristics [26], we have also studied the expression patterns of genes involved in different aspects of energy metabolism, which is associated with meat quality, especially the intramuscular fat (IMF) content. The *MyHC* expression patterns observed in the WPs and DPs were in agreement with the higher number of SDH-positive myofibers and the higher IMFL content (estimated using the oil red O staining intensity) observed in WPs than in DPs. These findings corroborate with previous studies reporting a positive correlation between the abundance of *MyHC-I* transcript and SDH activity [27] or the IMF content [12], and a negative correlation between *MyHC-IIb* and the IMF levels [26]. Based on these results, we can assume that the relative expression levels of individual MyHCs are correlated with the expression levels of lipid metabolism-related genes. PGC-1α exerts a clear impact on metabolism via its effects on many downstream target genes that themselves affect lipid metabolism in pigs, as established by Erkens et al. [17]. Similar findings were reported by authors who demonstrated that *PGC-1α* expression was higher in local Chinese pigs, together with higher *MyHC-I*, -*IIa* and -*IIx* transcript levels, higher IMF levels and superior meat quality than those of Landrace pigs [12,13]. We showed that the *PGC-1α* level was higher during the early postnatal period in the WPs than in the DPs but that the difference in expression between the breeds was not significant in the adult pigs. In both breeds, the relative *PGC-1α* expression level decreased during the first three weeks, during which time the myofibers underwent intensive contractile and metabolic specialization. The level stayed the same in the adult WPs, whereas the level increased after the first three weeks in the DPs. The latter result can be explained by the recent finding that PGC-1α plays a role in myoblast differentiation/maturation [16]. Additionally, the mRNA expression levels of lipogenesis (e.g., *PPARγ*)—and fatty acid uptake (e.g., *LPL*)—related genes were reported to be higher in local fatty pig breeds, such as the Rongchang breed in China, than in commercial Landrace pigs, whereas the expression level of the fatty acid oxidation-related gene *CPT-1B* was higher in Landrace pigs [13]. We did not find any significant differences in the mRNA expression levels of the enzymes examined in this study between the WPs and DPs of the same age, but we did note some age-related differences. This observation was rather unexpected, but it corroborated the results of Park et al. [3], who suggested that energy metabolism and the contraction speed could be uncoupled in myofibers. To summarize, metabolic characteristics of pig skeletal muscle SDH activity and the oil red O IMFL staining intensity showed a clear relationship with the contractile profile reflected by MyHC expression, whereas unexpectedly no correlation was found with the expression of genes involved in lipid uptake and utilization. 

## 5. Conclusions

The present study confirms that WPs exhibit faster postnatal myofiber differentiation/maturation than DPs directed toward the development of oxidative myofibers, which are desirable for meat quality. The histochemical analysis of SDH activity and IMFL staining results corroborate with the MyHC expression pattern. However, the mRNA abundance of the studied genes involved in lipid uptake and utilization did not differ between the wild and modern domestic pig breeds, suggesting that post-transcriptional modifications regulate the metabolic activity of these enzymes.

## Figures and Tables

**Table 1:** Information on the primers and predeveloped assays used for qPCR.

**MyHC Isoform**	**Gene**	**Primer Sequence (5′→ 3′)**	**Amplicon Length**
embryonal	*MyHC_embry_*	F: CGGGTCCTTCCCATCTGA R: GCAGCAGCCGTGAGAAATC P: FAM-CTGCCCGGCTTTGGTC	75
I	*MyHC-I*	F: CGTGCTCCGTCTTCTTTCCTT R: GAAGAAAGAGGCTCAAGCTGGAA P: FAM-CTGCTCTCAGGCCCC	60
2a	*MyHC-2a*	F: GTCCTGCTTTAAAAAGCTCCAAGAA R: AGAGGTCCCTCTTAGCAAGTGA P: FAM-CAGGCTGCATCTTC	73
2b	*MyHC-2b*	F: AACACTTTAAGTAGTTGTCTGCCTTGA R: AGGGCACTAGATGTACCTCTTATGT P: FAM-CCTGCCACCGTCTTC	76
2x	*MyHC-2x*	F: GCTTCAAGTTCTGCCCCACTT R: TGGCAGCCCAGTCAAAGAC P: FAM-CTTTGAGATGCAACCTTG	59
**Full Gene Name**	**Gene**	**Amplicon Length**	**Assay ID**
Peroxisome proliferator-activated receptor gamma, coactivator 1 alpha	*PGC-1α*	96	Ss03393114_u1
Peroxisome proliferator-activated receptor gamma	*PPARγ*	72	Ss03394829_m1
Lipoprotein-lipase	*LPL*	66	Ss03394612_m1
Carnitine palmitoyltransferase 1B	*CPT1B*	60	Ss03378792_u1
Eukaryotic ribosomal (r) 18S rRNA	*18S rRNA*	69	Hs03003631_g1

F = forward primer; R = reverse primer; P = probe.

## Supplementary Materials

The following are available online at http://www.mdpi.com/2076-2615/9/1/10/s1, Figure S1: Validation of the candidate internal control gene 18S rRNA. Table S1: ΔCt values of the studied transcripts in the longissimus dorsi muscle of wild pigs (WPs) and domestic pigs (DPs).
