# Supplementary material for: Age-Dependent Expression of MyHC Isoforms and Lipid Metabolism-Related Genes in the Longissimus Dorsi Muscle of Wild and Domestic Pigs
Source: Animals (Basel). 2018 Dec 28;9(1):10. doi: 10.3390/ani9010010 (PMC6357074; doi:10.3390/ani9010010)
Supplement: Supplementary file 1 [file animals-09-00010-s001.docx]

Supplementary Materials: Age-Dependent Expression of MyHC Isoforms and Lipid Metabolism-Related Genes in the Longissimus Dorsi Muscle of Wild and Domestic Pigs

Milka Vrecl ^1^, Marko Cotman ^1^, Matjaž Uršič ^1^, Marjeta Čandek-Potokar ^2^ and Gregor Fazarinc ^1,^ *

^1^ University of Ljubljana, Veterinary Faculty, Institute of Preclinical Sciences, Gerbičeva 60, 1000 Ljubljana, Slovenia; milka.vrecl@vf.uni-lj.si (M.V.); marko.cotman@vf.uni-lj.si (M.C.); matjaz.ursic@vf.uni-lj.si (M.U.);

^2^ Agricultural Institute of Slovenia, Hacquetova ulica 17, 1000 Ljubljana, Slovenia; meta.candek-potokar@kis.si

***** Correspondence: gregor.fazarinc@vf.uni-lj.si; Tel.: +386 1 477 9397

Received: 7 December 2018; Accepted: 19 December 2018; Published: 28 December 2018

**

**

**Figure S1.** Validation of the candidate internal control gene 18S rRNA. The effect of age/breed on 18S rRNA expression was validated using the following 2−ΔCt method as described in the Material and Methods sections: ΔCt = (Ctagex-Ctday1). WB: Wild pigs, DP: Domestic pigs. Values are shown as the means ± standard error of the mean (SEMs) and represent three independent experiments performed in triplicate. No statistically significant relationship was found between age/breed and 18S rRNA expression as derived from the *p* values for age, breed, and their combined effect (age x breed interaction) (two-way analysis of variance).

**Table S1.** ΔCt values of the studied transcripts in the longissimus dorsi muscle of wild pigs (WPs) and domestic pigs (DPs). The ΔCt values were derived using the comparative Ct method (ΔCt = Ct (target gene)—Ct (18S rRNA) and shown as the means ± SDs.

| **Gene name** | Δ**Ct (Day 1)** | | Δ**Ct (3 weeks)** | | Δ**Ct (2 years)** | |
| --- | --- | --- | --- | --- | --- | --- |
| **Transcript** | **WB (3)** | **DP (4)** | **WB (3)** | **DP (4)** | **WB (4)** | **DP (6)** |
| *MyHC-I* | 11.93 ± 0.35 | 12.99 ± 0.36 | 11.09 ± 0.37 | 12.23 ± 0.33 | 10.03 ± 0.45 | 11.30 ± 0.70 |
| *MyHC-IIa* | 10.20 ± 1.13 | 10.46 ± 1.23 | 9.77 ± 0.43 | 10.93 ± 0.92 | 9.18 ± 0.41 | 11.18 ± 0.66 |
| *MyHC-IIx* | 8.32 ± 0.14 | 9.59 ± 0.71 | 7.52 ± 0.26 | 8.05 ± 1.25 | 6.54 ± 0.60 | 7.68 ± 0.52 |
| *MyHC-IIb* | 17.70 ± 0.74 | 19.17 ± 1.83 | 9.19 ± 0.90 | 7.86 ± 0.62 | 8.50 ± 0.27 | 7.39 ± 0.46 |
| *MyHC_embry_* | 13.25 ± 1.93 | 13.39 ± 1.61 | 18.10 ± 0.90 | 15.46 ± 1.40 | 21.94 ± 0.38 | 22.51 ± 1.90 |
| *PGC-1α* | 17.18 ± 0.44 | 18.27 ± 0.35 | 18.19 ± 0.28 | 19.34 ± 1.03 | 18.41 ± 1.09 | 16.97 ± 0.67 |
| *PPARγ* | 19.01 ± 1.04 | 19.06 ± 0.41 | 19.71 ± 0.92 | 19.05 ± 0.53 | 20.44 ± 0.92 | 21.21 ± 0.26 |
| *LPL* | 12.84 ± 0.18 | 12.92 ± 0.59 | 14.25 ± 0.89 | 14.06 ± 0.78 | 14.28 ± 0.37 | 14.00 ± 0.52 |
| *CPT-1B* | 15.15 ± 1.13 | 15.59 ± 1.11 | 16.33 ± 0.99 | 16.41 ± 1.21 | 16.90 ± 0.42 | 16.87 ± 0.86 |

N is given in parentheses. ΔCt values around 20 indicate very low expression, because the average threshold cycle (Ct) in such cases was around 35. WB: Wild pigs, DP: Domestic pigs.
